# Predictors of Burnout in Parents of Children with Autism: The Importance of Physical Space

**DOI:** 10.3390/bs16060964

**Published:** 2026-06-10

**Authors:** Miraç Barış Usta, Şeyma Aker, Melis Elif Şenel, Meryem Macit Efe, Feride Burcu Taflan, Uygar Bayrakdar, Emrah Gulboy, Senay Kilincel, Oguzhan Kilincel

**Affiliations:** 1 Department of Child and Adolescent Psychiatry, Ondokuz Mayıs University, 55270 Atakum, Samsun, Türkiye; miracbaris.usta@omu.edu.tr (M.B.U.);; 2 Developmental Education Practice and Research Center (OGEM), Ondokuz Mayıs University, 55270 Atakum, Samsun, Türkiye; 3 Department of Child and Adolescent Psychiatry, Istanbul Aydin University, 34295 Küçükçekmece, Istanbul, Türkiye; 4 Department of Child Development, Istanbul Gelisim Universtiy, 34310 Avcılar, Istanbul, Türkiye

**Keywords:** balance of risks and resources, parental burnout, physical living space, social support

## Abstract

Parental burnout, characterized by emotional exhaustion and a sense of detachment, is a significant challenge for families of children with autism spectrum disorder (ASD). While traditional research often focuses on socioeconomic status, this study examines how physical living conditions and household characteristics influence burnout levels in the Turkish context. A relational survey model was conducted with 131 parents of children with ASD. Data were collected via online surveys using a Sociodemographic Data Form and the Maslach Burnout Inventory (MBI), which was adapted to the parenting role. Multiple linear regression analysis was applied to identify variables predicting total burnout scores. The analysis revealed that most sociodemographic variables, including income, education level, and parental age, did not significantly predict burnout. However, the “number of rooms in the house” was identified as the only statistically significant predictor (*p* = 0.033). Specifically, as the number of rooms increased, the total burnout scores of parents significantly decreased. The findings suggest that physical living space serves as a critical “spatial resource” and a protective factor against parental burnout. For families of children with ASD, having a private area for self-regulation is more decisive for mental health than economic status alone. Future support strategies should prioritize improving the quality of life through spatial arrangements and respite services.

## 1. Introduction

Autism spectrum disorder is a neurodevelopmental disorder generally characterized by impairments in social interaction and communication skills, as well as restricted interests and repetitive behaviors. The notion of a ‘spectrum’ reflects the wide variation in these characteristics, establishing that there is a broad range of possible expressions of autistic tendencies and strengths in individuals with ASD. Starting in early childhood and continuing throughout life, this condition can lead to significant limitations in individuals’ social, academic, and daily functioning. According to data from the Centers for Disease Control and Prevention (CDC), the prevalence of autism spectrum disorder (ASD) among 8-year-old children in the United States in 2022 was determined to be 32.2 per 1000 children (3.2%); this means that one in every 31 children received an ASD diagnosis (Centers for Disease Control and Prevention, 2025). Recent studies in Türkiye emphasize that increasing family resilience is a critical protective factor that reduces parental burnout in families with children with special needs. Furthermore, it has been observed that an increase in parents’ levels of hopelessness directly triggers feelings of burnout, thereby disrupting the resource–risk balance within the family (Yılmaz & Bahçalı, 2025).

Since ASD is a disorder that begins in early childhood and lasts a lifetime, it directly affects family life as much as the child’s daily life (Özsoy et al., 2006). Parents of children with ASD experience higher levels of stress compared with parents of typically developing children or children with different developmental problems. While navigating the unique needs of a child with ASD requires significant adaptation, it is crucial to recognize that parents and children frequently establish profound and deeply rewarding emotional bonds. The narrative of parental burnout should not be exclusively attributed to the child’s neurodivergent traits (Howard & Sedgewick, 2021). Various challenges in the care of children with ASD, such as the child’s cognitive inadequacies, irritability, dependency on the parent, hyperactivity, learning and language problems, the need for lifelong care, toileting and feeding issues, and the child’s constant desire to stay at home, can contribute significantly to increased stress in parents (Al-Oran & Khuan, 2021).

This long-standing stress can lead to burnout, defined by Maslach, which is characterized by loss of motivation, hopelessness, and decreased functionality in individuals (Maslach & Leiter, 2016). The core symptoms of parental burnout are an intense feeling of exhaustion related to the parenting role, a detachment focused only on physical care by avoiding emotional sharing in the relationship with children, and dissatisfaction with the parenting identity. The key point is that these symptoms contrast sharply with the parent’s past attitudes, and the parenting role is now seen as an unsustainable burden (Roskam et al., 2018). The core symptoms of parental burnout are an intense feeling of exhaustion related to the parenting role, a detachment focused only on physical care by avoiding emotional sharing in the relationship with children, and dissatisfaction with the parenting identity. This description of core symptoms, specifically intense exhaustion, emotional detachment, and dissatisfaction with the parental identity, is explicitly supported by the conceptual framework established by Roskam et al. (2018). In the care of children with ASD, continuity, unpredictability of behaviors, difficulties in accessing education and health services, social prejudices, and lack of social support are among the main risk factors for parental burnout (Karst & Van Hecke, 2012).

According to the “Balance between Risks and Resources” theory, parental burnout potentially arises from the imbalance between excessive parenting demands and limited parenting resources (Mikolajczak & Roskam, 2018). Individuals experiencing parental burnout face a decrease in the sense of accomplishment and happiness, emotional distancing from children, and disappointment in the parental role (Roskam et al., 2018). Additionally, negative consequences such as suicidal ideation, the urge to escape, alcohol and substance use, overeating, sleep disorders, and psychological escape behaviors can be observed (Mikolajczak et al., 2018).

Chronic parental burnout not only negatively affects the physical and mental health of parents and can lead to more serious mental problems such as stress, anxiety, and depression, but also directly reduces the quality of parenting and the quality of interaction with the child (T. R. Evans et al., 2023). It can increase conflict between spouses and lead to anger and irritability by lowering the parent’s threshold of tolerance. One of the most worrying consequences is the potential increased risk of neglect or verbal and physical violence toward children (Mikolajczak et al., 2019).

Early studies on parental burnout initially defined the concept inspired by general parenting stress and burnout models in professional life, focusing particularly on the chronic fatigue and emotional drainage experienced by parents of children with special needs since the 1980s (Pelsma et al., 1989). While research in this period highlighted the diagnostic characteristics of the child and the care burden as the primary source of burnout, the current literature has shifted its focus from only the child’s condition to the parent’s internal and external resources (Mikolajczak & Roskam, 2020). Recent studies conducted in 2024 and 2025 emphasize the decisive role of sociodemographic variables such as income level, education level, and household structure on burnout, while also highlighting the critical importance of parents’ internal resources such as psychological resilience, self-efficacy, and perceived social support in balancing risks (Findling et al., 2024). Current research conducted specifically in the Turkish sample shows that economic conditions and difficulties in accessing care services disrupt the resource–risk balance of parents, potentially increasing burnout levels, which, in turn, can directly affect parenting quality (Yılmaz & Bahçalı, 2025).

The aim of this study is to examine the burnout levels in parents of children with ASD and the relationship of this burnout with sociodemographic characteristics (age, gender, education level, income level, number of children, etc.). This study aims to contribute to the development of recommendations for supporting parents of children with ASD by filling the gaps in the literature.

## 2. Method

### 2.1. Research Design

This study was designed in the relational survey model, one of the quantitative research methods, aiming to determine the burnout levels of parents of children diagnosed with autism spectrum disorder (ASD) and the relationship of these levels with various sociodemographic variables. The sample of this research consisted of parents of children diagnosed with ASD who applied to the Ondokuz Mayıs University (OMU) Faculty of Medicine Child and Adolescent Mental Health and Diseases outpatient clinic and attended the Developmental Education Practice and Research Center (OGEM) within OMU. A total of 131 parents who participated in this study on a voluntary basis were included. Data were collected through online surveys prepared via Google Forms. The data in the study were collected using two primary tools.

#### 2.1.1. Sociodemographic Data Form

This form, developed by the researchers, includes personal information, such as the age, marital status, education level, and occupation of the parents, as well as comprehensive variables, such as the number of household members, the number of rooms in the house, the child’s age at diagnosis, school status, types of special education received (speech therapy, occupational therapy, sensory integration, etc.), and psychiatric medication treatment.

#### 2.1.2. Maslach Burnout Inventory (MBI)

The scale developed by Maslach and Jackson in 1981 was used to measure the burnout levels of parents (Maslach & Jackson, 1981). Validity and reliability studies of the scale in Turkey were conducted by Ergin and Çam (Ergin, 1992). The scale consists of a total of 22 items and three subdimensions: emotional exhaustion (9 items), depersonalization (5 items), and reduced personal accomplishment (8 items). Items were evaluated using a Likert-type scale ranging from 1 to 5.

In the scoring of the scale, items in the personal accomplishment subdimension (items 4, 7, 9, 12, 17, 18, 19, and 21) are reverse-scored. A high score in this subdimension indicates low burnout, while a low score indicates high burnout (a high reduction in personal accomplishment). In the emotional exhaustion and depersonalization subdimensions, higher scores indicate an increase in the level of burnout in the respective dimension.

Although the PBA is domain-specific, the MBI was utilized due to its long-standing validation in Turkey. Items were meticulously restructured as ‘my child’ and ‘care of my child’ to ensure contextual relevance, yielding a high internal consistency of 0.93 in this study. In accordance with the methodology suggested in pioneering studies in the literature, the scale items were restructured as ‘my child’ and ‘care of my child’ to make general burnout expressions specific to the parenting role (Duygun & Sezgin, 2003; Pelsma et al., 1989). The Cronbach’s alpha internal consistency coefficient of the scale was determined to be 0.93.

### 2.2. Statistical Analysis

Analysis of the data was performed using the IBM SPSS Statistics v22 program. Before the analysis, the data were checked, and necessary coding was performed; the total burnout score was calculated as the dependent variable. The total burnout score was obtained by summing the subdimension scores of the scale (emotional exhaustion + personal accomplishment + depersonalization). In line with the main purpose of the study, hierarchical multiple linear regression analysis) was applied to determine the variables predicting the total burnout score. Continuous variables were included directly in the model. Categorical variables were handled with the dummy variable approach suitable for regression analysis in SPSS, and one level for each categorical variable was determined as the reference category, with comparisons made against this reference level. Regression results were reported with the unstandardized coefficient (B), standard error (SE), standardized coefficient (β), t-value, *p*-value, and 95% confidence interval. The overall fit of the model was evaluated through R^2^ and adjusted R^2^ values.

### 2.3. Research Ethics

Ethical approval was obtained for this study from the Ondokuz Mayıs University Clinical Research Ethics Committee with the decision number 2025/141 (date: 22 April 2025). To address potential emotional distress arising from the survey’s focus on parental burnout, a debriefing page was included at the end of the questionnaire. This page provided participants with direct contact information for the Ondokuz Mayıs University Child and Adolescent Psychiatry outpatient clinic and psychological support hotlines, ensuring access to professional help if needed.

## 3. Results

### 3.1. Parent and Household Characteristics

The vast majority of the 131 parents participating in this study consisted of mothers (77.9%) and married individuals (90.1%). The mean age was found to be 37.72 ± 7.15 for mothers and 40.79 ± 8.82 for fathers. Regarding education level, high school graduation ranked first in both parent groups. It was determined that 84.7% of the mothers were not employed in any job, while 43.5% of the fathers worked as laborers. The average number of children in the families was 2.02 ± 0.93, and the average number of rooms in the residences was 3.79 ± 0.71 (Table 1).

The average age of the children within the scope of this study was 8.4 ± 4.14 years, and 74.0% of the children were male. It is observed that the vast majority of the children (87.0%) attended school; regarding the educational environment, special education classes (50.4%) were most frequently used, followed by inclusion classes (21.4%) and autism practice schools (21.4%). In the sample, 93.1% of the children received special education support, and 60.3% were actively undergoing psychiatric medication treatment (Table 2).

In terms of child-specific clinical attributes, the vast majority of the participants were receiving special education services (n = 122, 93.1%), and a substantial proportion (n = 79, 60.3%) were undergoing psychiatric medication treatment. Conversely, rates of hereditary psychiatric history were relatively low within the cohort; a parental history of psychiatric illness was documented in only 9.2% (n = 12) of cases, while the presence of another child with a psychiatric illness was reported in 6.1% (n = 8) of families (Table 3).

### 3.2. Descriptive Statistics Regarding Scale Scores

Descriptive statistics regarding the scores obtained by the participants from the parental burnout scale and its subscales are presented in Table 4. The mean total burnout score of the sample was determined to be 50.57 ± 9.26 (median = 51.0).

When the subscales of the scale were examined, the mean score for emotional exhaustion was determined as 13.85 ± 9.44, the mean score for depersonalization as 5.42 ± 4.53, and the mean score for personal accomplishment as 37.97 ± 7.19. These data generally reveal the distribution of the burnout levels and subscale scores of the parents in the sample (Table 4).

The results of the Spearman correlation analysis, conducted to determine the relationships between the scale’s total score and its subscales, are presented in Table 5.

According to the analysis results, highly significant positive correlations were found between the total burnout score and the subscales of emotional exhaustion (ρ = 0.909; *p* = 0.001) and depersonalization (ρ = 0.734; *p* = 0.001). Conversely, a significant negative correlation was observed between the total burnout score and the personal accomplishment (ρ = −0.655; *p* = 0.001) subscale.

When the relationships between the subscales themselves were examined, it was determined that there was a moderate positive correlation (ρ = 0.502; *p* = 0.001) between emotional exhaustion and depersonalization. Furthermore, the personal accomplishment dimension showed an inverse (negative) correlation with both emotional exhaustion (ρ = −0.545; *p* = 0.001) and depersonalization (ρ = −0.450; *p* = 0.001). These findings indicate that as parents’ levels of emotional exhaustion and depersonalization increase, their perceptions of personal accomplishment decrease.

Hierarchical Regression Analysis Results: A three-step hierarchical linear regression analysis was conducted to identify the variables predicting total burnout scores in parents of children with autism spectrum disorder (ASD). Prior to the analysis, diagnostic checks were performed, confirming that VIF values were below 3.38, indicating no multicollinearity issues among the variables. A summary of all models included in the analysis is presented in Table 6. Model 1 (Demographic): In the first step, variables including mother’s age, father’s age, and child’s age did not explain a significant portion of the variance in parental burnout (R^2^ = 0.001; *p* > 0.05). Model 2 (Socioeconomic): In the second step, the addition of education level, employment status, and the total number of children increased the explained variance to 2.8%; however, this increase was not statistically significant (Delta R^2^ = 0.027; *p* > 0.05). Model 3 (Spatial): In the final step, the “number of rooms in the house” was included in the model, bringing the total explained variance to 5.8% (R^2^ = 0.058). In the final model (Model 3), after controlling for demographic and socioeconomic factors, the “number of rooms in the house” emerged as the only significant predictor of parental burnout (B = −3.87, β = −0.25, t = −2.17, and *p* = 0.033). The negative coefficient (B = −3.87) indicates that as the number of rooms in the house increases, the total parental burnout scores decrease significantly. The 95% confidence interval for this finding, calculated as [−7.41, −0.32], does not include zero, confirming the statistical significance of the result (Table 7). The effects of all other demographic and socioeconomic variables on burnout remained non-significant (*p* > 0.05).

## 4. Discussion

This study aimed to examine the burnout levels in parents of children diagnosed with autism spectrum disorder (ASD) and the relationship of this process with sociodemographic factors from a holistic perspective. The fact that our model explained 5.8% of the variance indicates that parental burnout is governed by numerous complex factors not captured in this study, such as ASD symptom severity, the level of perceived social support, and internal psychological resilience (Weinberg et al., 2021). However, the primary aim of this research was to isolate and emphasize the role of ‘spatial resources,’ a factor frequently neglected in the literature. This low variance ratio supports the interpretation that while physical space is not a standalone solution, it is a critical component of a holistic support model. The data obtained within the scope of this research show that parental burnout is not limited only to individual or economic characteristics, but can also be affected by the physical living conditions of the family. In particular, contrary to many variables frequently emphasized in the literature, the fact that the number of rooms in the house plays a decisive role in burnout scores highlights the importance of spatial opportunities and personal space in protecting parents’ mental health.

This result is consistent with the ‘Balance between Risks and Resources’ (BR2) model, which defines parental burnout as an imbalance between excessive demands and limited resources, and emphasizes that physical living space is a critical ‘spatial resource’ for the parent (Mikolajczak & Roskam, 2018). The relationship found between the number of rooms and burnout in our study parallels current studies in the literature that emphasize the negative effect of limited living space on parental mental health. Specifically, Wauters et al. (2022) revealed that the inadequacy of physical space and the feeling of crowding within the home are fundamental stressors that trigger parental burnout in parents of children with chronic health problems (Wauters et al., 2022). Indeed, in a study conducted in a Turkish sample, it was found that a significant portion of families with children with disabilities found the physical conditions of their homes inadequate and experienced difficulties due to their residences being ‘narrow and small’ for their children’s needs (Özsoy et al., 2006). When combined with the fact that the number of rooms was the only significant variable in our study, this situation supports the interpretation that the spaciousness of the physical space where daily life takes place plays a more primary role in protecting the psychological well-being of parents than socioeconomic indicators. It is also worthwhile linking these findings about the importance of space to an interactional model of disability. As Shakespeare (2012) suggests, constructing an alternative account of disability based on an interactional or relational understanding reveals that disability results from an interplay of individual and contextual factors. In other words, people are disabled by society and by their bodies. From this perspective, the individual variation within ASD interacts with different factors within the environment, such as the availability of physical space highlighted in this study. This interaction can help to explain the wide variety of ways in which families manage the complex business of parenting, learning, and development (Shakespeare, 2012).

Research examining the effect of residential crowding on family dynamics in the literature has revealed that physical space limitations impair the psychological health of parents and increase intra-family tension (Solari & Mare, 2012). The limited physical living space can make it nearly impossible for the parent to find a ‘psychological break area’ where they can step away from the parenting role, even for a short time. Since the caregiving process for children with ASD inherently requires high levels of attention and emotional labor, parents need a private space where they can regulate themselves during the day. Indeed, it has been determined in the literature that the inadequacy of privacy and personal space within the residence can reduce individuals’ capacity to cope with stress and accelerates emotional exhaustion by creating ‘spatial stress’ (G. W. Evans, 2003). In particular, limited physical space can cause parents to experience more irritability and intolerance in their interactions with their children.

Behavioral characteristics, such as sensory sensitivities, temper tantrums, and excessive adherence to routines often experienced by children with ASD, make the importance of physical space within the home even more critical. During moments of loud reactions or sensory overload in the child, the absence of a separate room as a safe zone where both the child can calm down and the parent can distance themselves from these intense stimuli can escalate tension within the home. In the literature, the need for ‘quiet areas’ for the sensory regulation needs of children with ASD is emphasized; it is stated that the absence of these areas can increase the ‘state of alertness’ and chronic stress in parents (Kanakri et al., 2017; Mostafa, 2008; Schaaf et al., 2012). Indeed, in another study, it was revealed that spatial inadequacies and physical clutter in homes where children with ASD live are environmental factors that can directly escalate parents’ stress levels (Bagby et al., 2012).

Another important finding of our research is that classical sociodemographic variables such as income level, education level, and parental age, which are frequently reported to be closely related to parental burnout in the literature, did not create a statistically significant difference in burnout scores. This result contradicts many studies arguing that low socioeconomic status increases burnout (Karst & Van Hecke, 2012; Özsoy et al., 2006; Roskam et al., 2021). The fact that income level was non-significant in our study warrants deeper contextualization within the Turkish sociocultural and systemic framework. In Türkiye, children diagnosed with ASD are legally entitled to state-funded special education and rehabilitation services, regardless of household income. This universal access likely acts as a significant buffer against the financial strain typically associated with private therapeutic interventions. Furthermore, the prevailing collectivist family structure in Türkiye often entails close physical proximity to extended family members. This dynamic frequently provides informal respite care, emotional solidarity, and resource sharing, which can profoundly mitigate the direct impact of economic inadequacies on parental emotional exhaustion (Doyumğaç et al., 2026). Research arguing that parental burnout is more closely related to parental personality traits and cultural context rather than socioeconomic indicators points out that the effect of sociodemographic factors on burnout may remain limited (Kawamoto et al., 2018). Indeed, a recent study conducted in a Turkish sample also found that sociodemographic characteristics such as income level, educational status, and age of parents did not create a significant difference in burnout (Cici & Ozdemir, 2024). The lack of a significant difference in education level on burnout may be interpreted as the information advantage possessed by highly educated parents being balanced by the expectation of ‘perfect parenting’ and the psychological pressure developing accordingly, as emphasized in the literature (Sorkkila & Aunola, 2020). This situation suggests that the caregiving burden brought by having a child with ASD poses a similar risk for parents from all walks of life, independent of the family’s socioeconomic status. No statistically significant difference was found between the burnout levels of mothers and fathers in the study. This finding shows that the chronic stress and emotional burden created by caring for a child with ASD creates a similar effect on both parents, regardless of gender. Although some studies in the literature state that mothers are at higher risk (Olsson & Hwang, 2001), studies conducted in recent years emphasize that fathers also exhibit similar burnout levels with their more active participation in the child care process (Hastings, 2003). This can be explained by the intertwining of parenting roles in the modern family structure.

The high prevalence of unemployed mothers (84.7%) in our sample suggests that the majority of participants spend a significant portion of their daily lives within the residential environment. This prolonged exposure likely intensifies the impact of spatial constraints; for parents who are constantly at home, the lack of a ‘psychological break area’ or a private room for self-regulation can more rapidly deplete emotional resources and accelerate burnout.

The lack of a significant relationship between the child’s age and the time elapsed since diagnosis and burnout scores can be explained by the ‘adaptation process’. Over time, parents overcome the intense shock and uncertainty they experience in the first years after diagnosis and develop coping mechanisms for the child’s special needs (Lavee et al., 1996). This situation, referred to as the ‘healing effect of time’ in the literature, is associated with an increase in parents’ crisis management skills and their more effective access to social support networks (Gray, 2002). The non-significance of sociodemographic variables in this research may have resulted from the sample group exhibiting a similar structure as they were selected from applications within the same region. The participants having similar socioeconomic and education levels may have restricted statistical variation and masked the true effect of the variables.

Limitations: This study has some limitations. First, the cross-sectional design of this study limits the ability to reach definitive judgments regarding the causality of the relationship between the number of rooms and parental burnout. The cross-sectional design of this study limits our ability to draw definitive causal inferences regarding the relationship between the number of rooms and parental burnout. While our theoretical framework posits that limited physical space exacerbates burnout by depriving parents of a psychological break area, the possibility of bidirectional or reverse causality must be acknowledged. It is plausible that families experiencing lower baseline levels of burnout—potentially due to stronger internal resources or fewer co-occurring child behavioral challenges—may possess greater socioeconomic mobility, occupational stability, and psychological capacity to relocate to larger residences. Conversely, severe and chronic parental burnout may impede a family’s energy and financial ability to improve their physical living conditions. In future studies, it is recommended to use longitudinal designs, increase the representation of fathers, and include mediating variables such as psychological resilience in addition to the physical living space. Second, the fact that the data were collected through self-report scales raises the possibility that participants may have responded with social desirability concerns and the associated risk of bias. Additionally, the sociodemographically homogeneous structure of the sample and the fact that the vast majority of participants consisted of mothers restrict the generalization of the findings to the entire parent population. Additionally, the sample size (n = 131) is relatively small for a multi-variable regression analysis, which may restrict the statistical power of the results. Additionally, the predominance of mothers (77.9%) and the regional focus on Samsun limit the generalizability of these findings to fathers or to different sociocultural settings. Furthermore, while we focused on the ‘number of rooms,’ this study did not account for housing density (inhabitants per room) or qualitative environmental factors such as noise insulation and natural light, which are pivotal in environmental psychology and therapeutic architecture. Future research should incorporate these metrics alongside psychological mediators like marital satisfaction to provide a more holistic explanation of burnout.

Lastly, while focusing on the ‘number of rooms,’ we did not account for housing density or qualitative factors like noise insulation and natural light, which are pivotal in therapeutic architecture. Future research should incorporate these metrics to better define parents’ ‘psychological break area’.

## 5. Conclusions

This research has revealed that the burnout levels of parents of children with ASD can be closely related to the physical living space (number of rooms) within the home, rather than the sociodemographic variables such as income and education commonly discussed in the literature. The findings show that regardless of the parents’ socioeconomic means, undertaking the care of a child with ASD in a limited space can emotionally exhaust the parents and create a need for a ‘psychological break area’. Consequently, it is understood that economic support alone will not be sufficient in combating parental burnout; spatial arrangements that will increase the quality of life of families and social support systems, such as respite houses and day care centers, where parents can devote time to themselves, are of vital importance.

## Figures and Tables

**Table 1:** Parent and household characteristics.

Variable	Category/Statistic	n	%	Mean ± SD
Parent completing the form	Mother	102	77.9	
	Father	29	22.1	
Marital status	Married	118	90.1	
	Single	13	9.9	
Mother’s education level	Illiterate	1	0.8	
	Primary school graduate	16	12.2	
	Middle school graduate	17	13.0	
	High school graduate	53	40.5	
	Associate degree	17	13.0	
	Bachelor’s degree	22	16.8	
	Graduate degree	5	3.8	
Father’s education level	Illiterate	2	1.5	
	Primary school graduate	9	6.9	
	Middle school graduate	20	15.3	
	High school graduate	44	33.6	
	Associate degree	13	9.9	
	Bachelor’s degree	39	29.8	
	Graduate degree	4	3.1	
Mother’s occupation	Unemployed	111	84.7	
	White-collar	10	7.6	
	Laborer	4	3.1	
	Specialist	4	3.1	
	Blue-collar	2	1.5	
Father’s occupation	Laborer	57	43.5	
	White-collar	34	26.0	
	Blue-collar	23	17.6	
	Unemployed	11	8.4	
	Specialist	6	4.6	
Mother’s age (years)		131		37.72 ± 7.15
Father’s age (years)		131		40.79 ± 8.82
Number of rooms in house		131		3.79 ± 0.71
Total number of children		131		2.02 ± 0.93

Note: Categorical variables are given as n (%); continuous variables are given as means ± SD. Percentages were calculated after removing missing data for the relevant variable.

**Table 2:** Characteristics related to the child (school/education/treatment).

Variable	Category/Statistics	n	%	
Child’s gender	Male	97	74.0	
	Female	34	26.0	
Child’s school attendance status	Attending	114	87.0	
	Not attending	17	13.0	
Grade/classroom type	Special education class	66	50.4	
	Inclusion class	28	21.4	
	Autism practice school class	28	21.4	
	Not attending school	5	3.8	
	Individual home education	4	3.1	
Special education status	Receiving	122	93.1	
	Not receiving	9	6.9	
Psychiatric medication treatment	Receiving	79	60.3	
	Not receiving	52	39.7	
Child’s age (years)		131		8.4 ± 4.14

Note: Categorical variables are presented as n (%), and continuous variables are presented as means ± SD. Percentages were calculated after excluding missing data for the relevant variable.

**Table 3:** Clinical characteristics.

Variable	Category/Statistics	n	%
**Clinical characteristics related to the child**			
Psychiatric medication treatment	Receiving	79	60.3
	Not receiving	52	39.7
Special education status	Receiving	122	93.1
	Not receiving	9	6.9
**Family history of psychiatric illness**			
Is there another child with a psychiatric illness?	No	123	93.9
	Yes	8	6.1
Is there a psychiatric illness in the mother/father?	No	119	90.8
	Yes	12	9.2

Note: Categorical variables are presented as n (%). Percentages were calculated after excluding missing data for the relevant variable.

**Table 4:** Descriptive statistics.

Scale/Subscale	n	Mean ± SD	Median (IQR)
Total burnout score	131	50.57 ± 9.26	51.0 (43.0–56.5)
Emotional exhaustion	131	13.85 ± 9.44	12.0 (6.0–21.0)
Depersonalization	131	5.42 ± 4.53	5.0 (1.5–8.0)
Personal accomplishment	131	37.97 ± 7.19	40.0 (35.0–42.5)

Note: Means ± SD and medians (IQR) are reported for continuous variables.

**Table 5:** Correlations between subscales.

	Total Burnout Score	Emotional Exhaustion	Depersonalization	Personal Accomplishment
Total burnout score	1.0			
Emotional exhaustion	0.909 *	1.0		
Depersonalization	0.734 *	0.502 *	1.0	
Personal accomplishment	−0.655 *	−0.545 *	−0.450 *	1.0

Note: Correlations were calculated using Spearman’s ρ (rho). * *p* < 0.05.

**Table 6:** Summary of hierarchical regression analysis models.

Model	Variables	R^2^	Adj. R^2^	ΔR^2^	F_change_	*p*
**Model 1**	Demographic variables (mother’s age, father’s age, and child’s age)	0.001	−0.021		0.043	0.988
**Model 2**	+ socioeconomic variables (education level, employment status, and the total number of children)	0.028	−0.017	0.027	1.171	0.325
**Model 3**	+ number of rooms in the house	**0.058**	**−0.004**	**0.03** **0**	**4.708**	**0.033** *

Note. n = 131; R^2^: coefficient of determination; Adj. R^2^: adjusted coefficient of determination; ΔR^2^: change in coefficient of determination; F_change_: F-test statistic for change in R^2^; * *p* < 0.05.

**Table 7:** Coefficients and confidence intervals for the final model (Model 3).

Variable	B	SE	β	t	*p*	%95 CI
Constant	92.29	22.93	-	4.02	0.001	[46.76, 137.82]
Mother’s age	−0.29	0.32	−0.19	−0.91	0.368	[−0.93, 0.35]
Father’s age	0.32	0.23	0.26	1.44	0.154	[−0.12, 0.77]
Child’s age	−0.33	0.38	−0.12	−0.86	0.391	[−1.07, 0.42]
Number of rooms in the house	**−3.87**	**1.79**	**−0.25**	**−2.17**	**0.033** *	**[−7.41, −0.32]**
Education level (university+)	−2.61	5.37	−0.08	−0.49	0.628	[−13.27, 8.05]
Employment status (full-time)	−4.73	6.07	−0.16	−0.78	0.438	[−16.8, 7.33]
Total number of children	0.84	1.61	0.07	0.52	0.602	[−2.35, 4.03]

Note. n = 131; R^2^ = 0.058; * *p* < 0.05; B: unstandardized coefficient; SE: standard error; Beta: standardized coefficient; t: t-test statistic; *p*: *p*-value; 95% CI: 95% confidence interval; education level (1: university+; 0: other); employment status (1: full-time; 0: other).

## Data Availability

The data presented in this study are available upon request from the corresponding author. The data are not publicly available due to privacy and ethical restrictions, as they contain sensitive information that could compromise the anonymity of the participants.
